# The Impact of Immune Response on HTLV-I in HTLV-I-Associated Myelopathy/Tropical Spastic Paraparesis (HAM/TSP)

**Published:** 2013-03

**Authors:** Houshang Rafatpanah, Reza Farid Hosseini, Seyed Hassan Pourseyed

**Affiliations:** 1 Inflammation and Inflammatory Diseases Research Centre, Mashhad University of Medical Sciences, Mashhad, Iran; 2 Allergy Research Centre, Mashhad University of Medical Sciences, Mashhad, Iran

**Keywords:** Human T lymphotropic virus type I (HTLV-I), HTLV-I-associated myelopathy/tropical spastic paraparesis (HAM/TSP), Immune response

## Abstract

Human T lymphotropic virus type I (HTLV-I) is a retrovirus which is associated with adult T cells leukaemia (ATL) and HTLV-I-associated myelopathy/tropical spastic paraparesis (HAM/TSP) in a minority of HTLV-I-infected individuals. It is not clear why a minority of HTLV-I-infected individuals develop HAM/TSP and majority remains lifelong carriers. It seems that the interaction between the virus and the immune response plays an important role in HTLV-I-associated diseases. Although the role of the immune response in HTLV-I pathogenesis is not fully understood, however it seems that the efficacy of the immune response which is involved in controlling or limiting of viral persistence determines the outcome of HTLV-I-associated diseases. Here we discuss the role of innate and adaptive immune response and also the risk factors contribute to the observed differences between HAM/TSP patients and asymptomatic HTLV-I carriers.

## Introduction

Human T-cell leukaemia virus type I (HTLV-I) is a type C retrovirus, which belongs to the Deltaretrovirus genera of the Orthoretrovirinae subfamily, first identified 30 years ago from peripheral blood samples of a patients with cutaneous T-cell lymphoma (1). The virus is associated with two main types of diseases; adult T-cell leukemia (ATL) and a chronic inflammatory disease named HTLV-I-associated myelopathy (HAM)/tropical spastic paraparesis (TSP) (2-3). HTLV-I is also associated with other inflammatory diseases such as arthritis, uveitis, dermatitis, lymphadenitis and Sjo¨gren’s syndrome (4). 

Although, it is estimated 10-20 million individuals worldwide are infected with HTLV-I, the prevalence of the virus in the general population remains unknown (5-6). HTLV-I infection is endemic in the southwest of Japan, the Caribbean islands, Central and South America and West Africa (7-11). We have previously reported that HTLV-I infection is prevalent in north east of Iran, Mashhad and Sabzevar (12-14) and these cities are considered as the new endemic regions of the disease. 

The precise mechanism of the development and pathogenesis of HAM/TSP among fewer than 3% of HTLV-I carriers still remains unknown. Furthermore, the etiology of why fewer than 2% of asymptomatic HTLV-I carriers develop HAM/TSP or ATL, whereas the others remain lifelong carriers, is poorly understood. Studies have demonstrated that viral factors and genetic background may influence the outcome of HTLV-I infection (15-17). There is no clear evidence for association between HTLV-I variants and susceptibility to HAM/TSP in the carriers (18). Although, it had been suggested that an amino acid substitution in the Tax protein increases HAM/TSP risk in HTLV-I carriers; studies by Mahieux *et al* showed that mutation in the Tax gene was linked to HTLV-I subtype rather than the risk of HAM/TSP (19). More recently, Furukawa *et al* reported that a subtype of the Tax gene was more frequently observed in patients with HAM/TSP compared with HTLV-I carriers (17).

Accumulated data suggests that the interaction between the virus and the host plays a critical role in determining the risk of HTLV-I-associated diseases among HTLV-I carriers. The quality of immune response, especially cytotoxic T lymphocytes (CTL) are critical in controlling or limiting and also the efficacy of the host response to HTLV-I.

In this study, we review and update insights relevant to the immune response to HTLV-I in HAM/TSP patients. The role of HTLV-I in ATL will not be discussed here. 

*Immune response to HTLV-I*

***Innate immune response***

*In vivo* studies have shown that the HTLV-I provirus is mainly detected in CD4+ T lymphocytes (20). Infection of CD8+ T cells and B lymphocytes has also been reported (21-23). Thus, the adaptive immune cells seem to be the main target for HTLV-I *in vivo*.

The innate immune cells such as monocytes, macrophages and dendritic cells (DCs) are also shown to be infected *in vivo* and *in vitro *(21, 24-27). It has been demonstrated that both myDCs and pDCs are infected by HTLV-I *in vivo *(27). DCs and pDCs constitute less than 1% of peripheral blood cells, therefore these cells do not significantly contribute to the viral replication; however, their infection might result in dysfunctional immune response. 

It has been illustrated that both myDCs and pDCs are infected *in vitro *by cell-free HTLV virions and then the infected cells might transmit the virus to T cells (28). It is not fully understood whether HTLV-I-infected DC are infected through cell-to-cell contact or by cell-free virus *in vivo* (29). DC-Specific intercellular adhesion molecule-3-grabbing non-integrin (DC-SIGN) is a C-type lectine receptor, which is present on DC, with a pivotal role for the infection of DCs and transmission of the HTLV-I virus to T lymphocytes (30). Impaired IFN production has been shown in pDCs of HTLV-I-individuals, which is associated with high HTLV-I proviral load. This finding may suggest that the virus employs evasion mechanisms to inhibit the ability of IFN production by DC which may lead to facilitate virus spread (31).

Monocytes and macrophages might also act as HTLV-I reservoir *in vivo. *HTLV-I tax RNA expression has been confirmed in the RNA of monocytes, suggesting the virus has a broad host range *in vivo*.Monocyte lineage such as tissue macrophages might act as a virus reservoir *in vivo* (21). Infection of monocytes and monocyte-derived cells might have a role in the context of mother-to-child HTLV-I transmission. As far as macrophage constitute most of breast milk cells compared to T cells, prolonged breastfeeding could lead to viral transmission (29).

Natural killer cells (NKCs) act as critical regulators of innate and adaptive immunity which contribute to susceptibility and/or health protection (32). The role of NKCs response to HTLV-I has not been fully studied; however, patients with HAM/TSP represent significantly decreased NKCs frequency and activity compared with HTLV-I carriers (33-34). Although earlier work reported that activity and frequency of NK or NK-like cell response in HAM/TSP patients did not correlate with proviral load (33-35), Vine *et al*, 2004 indicated high levels of NKG2D gene expression, which served as a primary recognition receptor on NK cells, and a costimulatory molecule on CTLs were linked with HTLV-I low proviral load. It seems that both populations reduce the HTLV-I proviral load through cell-meditated lysis (36). 

In addition to T cells, NK cells also undergo significant spontaneous proliferation in both HTLV-I asymptomatic carriers and HAM/TSP patients in *ex vivo*. The spontaneous NK cell proliferation correlates with HTLV-I proviral load (37). The number of circulating NK T cells (NKT) is lower in HAM/TSP and ATLL patients than uninfected individuals (38). Low proliferation rate and reduced levels of perforin production in response to *ex vivo *stimulation in NKT cells in HTLV-I infected individuals suggest the impaired function of such cells (39). 

*Adaptive immune response*

***Humoral immune response***

The diagnosis of HTLV-I infection is based on the detection of specific antibodies by screening tests such as enzyme linked immunosorbent assay (ELISA) or particle agglutination. The reactive results should be confirmed by confirmatory methods such as Western blot (WB) or polymerase chain reaction (PCR). In Western blot, the reactive samples are tested against the products of HTLV-I such as gag (p19 or p24) and env (gp21 or gp46) gene products (40-41). 

After HTLV-I infection, antibodies to core, envelope and tax protein in serum appear within 30 to 60 days after primary HTLV-I infection; antibody to gag proteins predominant with anti-p24 generally appears before anti-p19 antibodies. Antibody to p-21 envelope protein frequently appears before gp46 antibodies. Anti tax antibodies are the latest antibodies that appear in the time course of seroconversion (42). Higher titres of specific IgG and IgA HTLV-I antibodies in sera and CSF in patients with HAM/TSP compared with asymptomatic HTLV-I carriers has been reported. In addition, the high titre of HTLV-I antibody significantly correlates with HTLV-I proviral load. Western blot analysis has shown that anti-HTLV-I IgM antibodies are present in most HAM/TSP patients and a few HTLV-I carriers. This indicates that continuous replication of HTLV-I is occurred in HAM/TSP patients (43-44). Most patients with HAM/TSP have oligoclonal IgG bands mainly in the CSF, which may be found in the serum. These bands are against p24 antigen (45). 

Real time polymerase chain reaction (PCR) also allows quantifying HTLV-I proviral load which is expressed as the number of HTLV-I DNA copies per fixed number of peripheral blood mononuclear cells (PBMC). Proviruses of HTLV-I are clonally integrated in ATL patients, but randomly integrated in HAM/TSP patients. The efficiency of HTLV-I replication is defined as the level of proviral DNA integrated in random PBMCs population or the proportion of PBMCs that carry a HTLV-I provirus (41, 46). HTLV-I proviral DNA is higher in HAM/TSP patients than HTLV-I carriers (47). The percentage of PBMC infected in HAM/TSP patients varies from 3% to 15% (48). The amount of HTLV-I proviral DNA in HAM/TSP can range from 2 to20 copies per 100 PBMCs, while in carriers it is 0.04 to 8 copies per 100 PBMC. Moreover, the amount of HTLV-I proviral DNA in patients with HAM/TSP is higher than HTLV-I carriers (49); however, 50% of asymptomatic carriers also have a high proviral load, which may suggest that measurement of this marker is not valuable for differentiation between patients with HAM/TSP and carriers (50). 

High proviral load and the presence of IgM antibodies and high titres of IgG and IgA antibodies to HTLV-I proteins appear to distinguish HAM/TSP patients from HTLV-I carriers (44). More recently, plasma proteome analysis in patients with HAM/TSP has shown that high concentrations of b2-microglobulin and Calgranulin and low concentration apolipoprotein A2 concentrations are associated with HAM/TSP (50). 

*HTLV-I specific CD8+ CTL response*

Cytotoxic T lymphocytes are a subset of T cells that recognise and kill their targets through the major histocompatibility complex (MHC). The majority of CTLs express CD8 molecules, whereas they rarely express CD4 molecule. Both CD4+ and CD8+ CTLs are able to eliminate infected cells and are important for recovery from viral infection (51).

Studies have shown that both HAM/TSP patients and asymptomatic carriers have circulatory HTLV-I specific CTLs. These CTLs are human leukocyte antigen (HLA) class I or class II restricted which recognise varieties of HTLV-I epitopes such as Tax, Rex and env proteins (52-53). Antigenic stimulation of HTLV-I infected cells *in vitro *generates HTLV-I specific CD4+ T cell lines. Most of these cells are HLA class II restricted and exhibit cytotoxic activity. Although these CTLs recognise the HTLV-I envelope protein between amino acids 196 and 209, their frequency in the circulation is low (53).

HTLV-I specific CD8+ CTLs with high frequency are obtained from the peripheral blood and CSF of patients with HAM/TSP, while the frequency of these cells in asymptomatic HTLV-I carriers is much lower or absent (54). This finding suggests that CD8+ CTLs might play a critical role in the pathogenesis of HAM/TSP by destruction of HTLV-I infected cells in the CNS. In contrast, some groups have reported that such specific CTLs (CD4+ and CD8+) are also present in HTLV-I asymptomatic carriers (55-56). Daenke *et al* found that there is no difference between HAM/TSP patients and healthy carriers in the frequency of specific CD8+ CTLs (57).

Most HTLV-I-specific CD8+ CTLs recognise the Tax protein (Tax11-19) in association with HLA-A2 allele. The tetrameric MHC-peptide complex technique identified that HAM/TSP patients carried a significantly higher number of CD8+ T cells specific for the HTLV-I Tax11-19 peptide in PBMC and CSF compared with the carriers (58). Bieganowska *et al* demonstrated that circulating CD8+ Tax11-19- reactive T cells were also found at high frequency in HAM/TSP patients. These cells exhibit migratory capacity and express different chemokine and cytokine receptors such as CXCR3, IL-8 receptor A and B (CXCR1 and CXCR2), CCR5 and the IL-2R β-chain (59). The frequency of HTLV-I Tax specific CD8+ with the characteristics of memory and/or effector cells is much higher in patients with HAM/TSP than HTLV-I carries. There is a direct relation between HTLV-I proviral load and the frequency of these cells in HAM/TSP patients, which might suggest Tax-specific cytotoxic response is promoted by proviral load (60). 

The high frequency of HTLV-I-specific CD8+ CTLs in PBL and CSF is responsible for the production of proinflammatory cytokines. Patients with HAM/TSP have high levels of peripheral blood CD8+ T cells that produce intracellular IFN-γ. It has been demonstrated that HTLV-I-specific CD8+ clones derived from HAM/TSP patients secret IFN-γ, TNF-α, IL-16, macrophage-inflammatory protein-1α (MIP-1α) and 1β (MIP-1β), and matrix metalloproteinase-9 (MMP-9) (61). 

Using intercellular cytokine detection Kubota *et al* shows that in HAM/TSP patients circulating HTLV-I-specific CD8+ lymphocytes produce proinflammatory cytokines such as IL-2, TNF-α and IFN-γ. The proportion of these cytokine expressing HTLV-I-specific CD8+ cells in total CD8+ T cells is extraordinary high (4.9% in HAM/TSP and 0.4% in HTLV-I carriers). These specific CD8+ T cells could promote inflammatory responses to HTLV-I in HAM/TSP (62). The proportion of HTLV-I-specific CD8+ lymphocytes, which produces IFN-γ ispositively correlated with the proviral load in PBMC of the HAM/TSP patients, but not with seropositive controls. The data could suggest that the CTLs immune response is driven by proviral load and enhances inflammatory responses to HTLV-I in HAM/TSP patients (63). 

The role of HTLV-I-specific CD8 T cells response in HTLV-I infection is not fully understood. It is still a matter of debate whether these cells contribute to the inflammatory and demyelinating process of HAM/TSP or whether the dominant effect of these cells *in vivo* is protective against disease through killing HTLV-I infected cells. It has been suggested that patients with HAM/TSP have a high frequency of HTLV-I-specific CD8 T cells with poor lytic capacity, whereas in the carriers, the frequency of such cells is lower, but they exhibit higher lytic capacity (64). Genetic factors such as MHC class I alleles, HLA-A*02 and HLA-Cw*08 are associated with a lower proviral load and a lower risk of HAM/TSP, suggesting restricted CTL to these alleles are efficient at killing HTLV-I infected cells. 

*T helper response*

CD4+ T cell response has a key role in driving CD8+ T-cell responses against viral infection *in vivo*. The most common antigen which is recognized by CD4+ T cells is the env protein, however other antigen such as gag and pol are also recognized by these cells (65-67). 

The frequency of HTLV-I specific CD4+ T cells is higher in HAM/TSP patients than HTLV-I carriers with a similar proviral load (65, 68). Furthermore, it has been suggested that there is a higher frequency of IFN-γ, TNF-α, and IL-2 production by CD4+ T cells in HAM/TSP patients than the carriers with a similar proviral load (65, 69). Immunogenetic association studies have reported that CD4+ T cells are important in initiating and causing HAM/TSP in different populations. Possession of HLA DRB*0101 in both Japanese and Iranian patients, who recognize the epitope of HTLV-I Env gp21 ,increases the risk of HAM/TSP in HTLV-I carriers (70-73). 

*Role of cytokines in HTLV-I infection*

*In vivo* studies by Tendler identifyalteration in host gene expression and cytokine production accompanies HTLV-I infection. The mRNA expression of TNF-α, IFN-γ and IL-1β is up regulated in HAM/TSP and HTLV-I carriers. This up-regulation is accompanied by an elevation of TNF-α and IL-1β exclusively in the sera of HAM/TSP patients (74). The high frequency of HTLV-I-specific CD8+ CTL in PBL and CSF is responsible for the production of proinflammatory cytokines. Patients with HAM/TSP have high levels of peripheral blood CD8+ T cells that produce intracellular IFN-γ. It has been demonstrated that HTLV-I-specific CD8+ clones derived from HAM/TSP patients secret IFN-γ, TNF-α, IL-16, macrophage-inflammatory proein-1α (MIP-1α) and 1β (MIP-1β), and matrix metalloproteinase-9 (MMP-9) (61). 

Using interacellular cytokine detection, Kubota *et al* shows that in HAM/TSP patients circulating HTLV-I-specific CD8+ lymphocytes produce proinflammatory cytokines such as IL-2, TNF-α and IFN-γ. Furthermore, IFN-γ is produced by Tax-specific CD8+ T cells when co-cultivated with autologus CD4+ T cells. The proportion of these cytokine expressing HTLV-I-specific CD8+ cell in total CD8+ T cells is extraordinary high (4.9% in HAM/TSP and 0.4% in seropositive asymptomatic carriers). These specific CD8+ T lymphocytes may promote inflammatory responses to HTLV-I in HAM/TSP (62). The same group also shows that the proportion of HTLV-I-specific CD8+ lymphocytes, which produce IFN-γ, positively, correlates with the proviral load in PBMC of the HAM/TSP patients, but not with seropositive controls. The data again suggests that the CTL immune response is driven by proviral load and enhances inflammatory responses to HTLV-I in HAM/TSP patients (63). Production of IFN-γ by HTLV-I infected CD4+ T cells with migratory phenotype may induce potentially harmful nonspecific inflammatory responses in the CNS (75).

*Role of regulatory T cells in HTLV-I infection*

CD4+ T cells are divided into two major categories, effector and regulatory T cells (Tregs). Effector T cells induce activation of immune responses by secreting pro-inflammatory cytokines whereas regulatory T cells, which express the transcription factor Foxp3, play a critical role in maintaining immune system homeostasis by both cell-contact dependent and independent mechanisms (76-77). *In vivo* studies have shown that HTLV-I infects CD4+CD25+ T cells that constitute the majority of the Foxp3+ Tregs (78-79). 

It has been suggested that the percentage of Foxp3+ Tregs in CD4+CD25+ cells in HTLV-I carriers and healthy controls is higher than this percentage in patients with HAM/TSP (78, 80); while in HTLV-I carriers the percentage of Foxp3+ cells in the CD4+ population is lower than this percentage in patients with HAM/TSP (81-83). 

Lately, it has been reported that patients with HAM/TSP exhibit significant reductions in Foxp3 expression and Treg cell function in CD4+CD25+ T cells (84). Reduced expression of CTL antigen-4 (CTLA-4) and glucocorticoid-induced tumor necrosis factor receptor-related protein (GITR) have also been demonstrated on the CD4+CD25+ T cells of the patients with HAM/TSP (78, 85). Interestingly, *in vivo* studies suggest that overexpression of HTLV-I Tax decreases the expression of Foxp3 and prevents Treg cells suppressive function. The frequency of HTLV-I negative Foxp3+CD4+ cells positively correlates with proviral load, while there is a negative association between CTLs’ activity and the frequency of these cells. This suggests that an increase in the number of HTLV-I negative Foxp3+CD4+ Tregs has a pivotal role in the efficiency of T cell mediated immune control of the virus in HTLV-I infected individuals (64).

It has been discovered that the chemokine receptor CCR4 is expressed on HTLV-I-infected leukemia cells in patients with ATL (86). Both Treg and Th2 cells in healthy individuals selectively express CCR4/ Also,the analysis of Foxp3 expression in CD4+CD25+CCR4+ T cells of HAM/TSP patients has shown that the frequency of the Foxp3– population has significantly increased in CD4+CD25+CCR4+ T cells (87). Notably, CD4+CD25+CCR4+ T cells of patients with HAM/TSP have shown an elevated amount of IFN-γ expression parallel with decreased expression of IL-4, IL-10, IL-17 and Foxp3 compared with healthy controls. These findings have proposed that this subpopulation is associated with the activity and severity of disease (87). Araya *et al* called this IFN-γ+Foxp3–CD4+CD25+CCR4+ T cell subsets THAM cells (84). 

**Figure 1:**
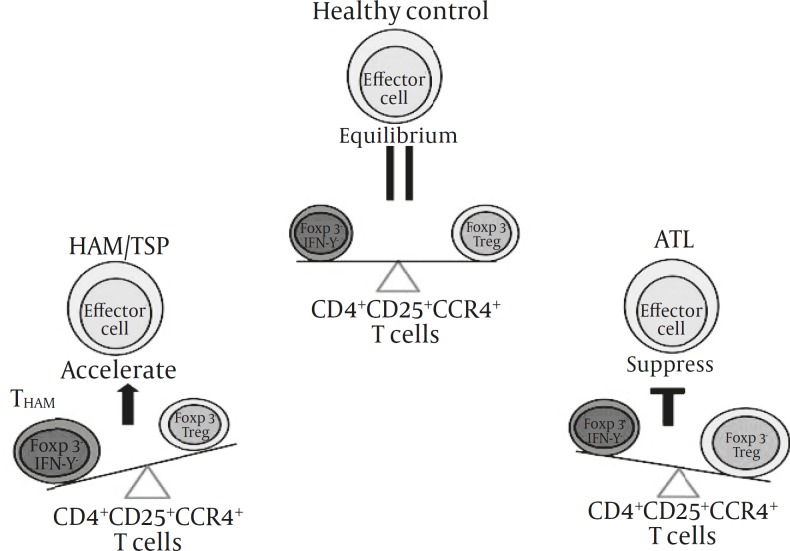
T_HAM_/Treg ratios in CD4+CD25+CCR4+ T cells determine the immune response differences in patients with HAM/TSP and ATL (adapted from reference 84

 In HAM/TSP and ATL patients, most of CD4+CD25+CCR4+ T cells are infected with HTLV-I (87-88) and the ratio of THAM cells (CCR4+Foxp3– with IFN-γ production) to Treg cells (CCR4+Foxp3+ with no cytokine production) in the CD4+CD25+CCR4+ T cell subset are higher in HAM/TSP patients compared with ATL patients (87). Therefore, it can be concluded that the difference in the ratios of THAM/Treg subpopulations in HTLV-I-infected T cells might determine the consequences of different immune responses between HAM/TSP and ATL patients (Figure 1).

## Conclusion

Accumulated data suggests that the vast majority of HTLV-I-infected individuals remain carriers and HTLV-I is not sufficient to cause HAM/TSP. Therefore, the differences in the efficiency of the immune response to HTLV-I infection and the genetic background among HTLV-I carriers could be crucial for determining the pathogenesis of HTLV-I infectious diseases. Both CTL response and regulatory T cells have critical roles in the outcome of HTLV-I-associated diseases. The CTL response in asymptomatic carriers is more efficient than CTL response in HAM/TSP patients, leading to more powerful predictive role in viral replication and spread. Furthermore, infection of regulatory CD4^+^CD25^+^CCR4^+^ T cells might also determine the outcome of different immune responses in HTLV-I infection.
